# Spontaneously Conversion from Film to High Crystalline Quality Stripe during Molecular Beam Epitaxy for High Sn Content GeSn

**DOI:** 10.1038/s41598-020-63152-y

**Published:** 2020-04-09

**Authors:** Nan Wang, Chunlai Xue, Fengshuo Wan, Yue Zhao, Guoyin Xu, Zhi Liu, Jun Zheng, Yuhua Zuo, Buwen Cheng, Qiming Wang

**Affiliations:** 1 State Key Laboratory on Integrated Optoelectronics, Institute of Semiconductors, Chinese Academy of Sciences, Beijing, 100083 P. R. China; 2 Center of Materials Science and Optoelectronics Engineering, University of Chinese Academy of Sciences, Beijing, 100049 China

**Keywords:** Silicon photonics, Phase transitions and critical phenomena, Surfaces, interfaces and thin films, Sensors and biosensors

## Abstract

Two series of Ge_0.8_Sn_0.2_ samples were grown on Ge buffered Si substrate by molecular beam epitaxy (MBE) to investigate the influence of growth temperature and film thickness towards the evolution of surface morphology. A novel phenomena was observed that the Ge_0.8_Sn_0.2_ film was segregated and relaxed by the formation of GeSn stripes on the film. Under specific growth condition, the stripes can cover nearly the whole surface. XRD, TEM, AFM, PL and TEM results indicated that the stripes are high quality single crystalline GeSn with Sn content around 5%. The formation of GeSn stripes proposes an effective strategy to fabricate high crystalline quality GeSn stripe on Si, where the Ge_0.8_Sn_0.2_ film serves as precursor and the segregated Sn works as catalyst droplets. This technique has great potential for future optoelectronic and microelectronic applications.

## Introduction

GeSn is a promising group IV material and has attracted more and more attention due to its great potential in high performance Si-based electronics and optoelectronics^1–3^. With the increase of Sn content, the bandgap is narrowed and transited to be direct^4,5^, which makes GeSn an attractive material for Si-based detector^6–8^ and light source^9–11^. Moreover, the incorporation of Sn atom into the Ge crystal can help to reduce the effective mass of the carriers and thus improve the carrier mobility^12^, making GeSn a competitive candidate as the channel material for high performance metal-oxide-semiconductor field-effect transistors (MOSFETs)^13–15^. All these properties are highly desirable in view of establishing a monolithic integration of Si-based optoelectronic components and logic devices^16^.

However, the application of GeSn material faces great challenges in the growth of GeSn film on Si and Ge substrate. First, different from the miscible Si and Ge system, the equilibrium solid solubility of Sn in Ge is less than 1%^17^. The Sn easily segregates during growth due to the lower surface energy compared to Ge^18^. Metastable growth at low temperature (100–400 °C) is required, but low growth temperature would enhance the density of point defects (vacancies or interstitials) and break the epitaxial structure^19^. Second, there is a large lattice mismatch between Ge, Si and α-Sn, for GeSn layer grown above the critical thickness, the film relaxes due to the formation of misfit dislocations at the interface. Continuous epitaxy may lead to the accumulation of defect, causing the segregation of Sn^20^. The defect-induced segregation of Sn during epitaxial progress is a complex problem, and understanding the mechanism behind can be useful for optimizing the growth technique, and then improve the crystalline quality.

Previously, GeSn films with Sn content up to 18% were synthesized^20,21^ to investigate the surface evolution and Sn segregation mechanism, and confirmed that the excessive growth temperature and the stain relaxation of film would lead to the segregation of Sn. It is found out that the segregated Sn may stay still^20^ or move and leave etching trace^21^or Sn wires^22,23^ on the surface due to the different Sn content, growth temperature, and substrate. In this work, in order to systematically investigate the influence of growth temperature and film thickness towards the evolution of surface, two series Ge_0.8_Sn_0.2_ samples (Table 1) were grown on Ge buffered Si substrate by molecular beam epitaxy (MBE). A new phenomena was observed that GeSn stripes would form on the surface at specific growth temperature after the film got relaxed, and a positive proportion was confirmed between the growth temperature and the occupation area of GeSn stripes. Under certain growth temperature, the stripes covers nearly the whole surface of the film. XRD, TEM, AFM and PL tests are performed and their results indicate that the stripes on Ge_0.8_Sn_0.2_ film are high quality single crystalline GeSn with 5% Sn content, and the stripes are formed by the migration of segregated Sn on Ge_0.8_Sn_0.2_ film driven by the Gibbs energy gap between the Ge_0.8_Sn_0.2_ film and GeSn stripes. These above results indicate that for high Sn content GeSn films, the over critical thickness growth may also cause the segregation of Sn on the surface. However, the segregated Sn may migrate on the surface and act as catalyst droplets for Ge_0.95_Sn_0.05_ stripes production. Under specific growth condition, the Ge_0.95_Sn_0.05_ stripe could cover nearly the whole surface of Ge_0.8_Sn_0.2_ film, indicating that Ge_0.8_Sn_0.2_ film spontaneously convert to Ge_0.95_Sn_0.05_ stripes with the catalyst of segregated Sn. This is a phenomena can help us understand how Sn and Ge atom interact during epitaxy, and can be used for producing GeSn stripes with high crystalline quality, which has extensive application in Si-based nanoelectronics and optoelectronics.

**Table 1:** Parameters for the samples of series A and B.

Series A (50 nm)	A1	A2	A3	A4	A5
Thickness (nm)	50	50	50	50	50
Xsn (%)	20	20	20	20	20
Growth Temperature (°C)	155	160	165	170	175
Series B (170 °C)	B1	B2	B3	B4	A4
Thickness (nm)	7	18	29	40	50
Xsn (%)	20	20	20	20	20
Growth Temperature (°C)	170	170	170	170	170

Growth parameters of GeSn samples grown on Ge buffered Si substrates. In this paper, two series of GeSn samples were prepared. In series A, 50 nm GeSn films with 20% Sn content were prepared at epitaxial temperature varied from 155 to 175 °C; in series B, GeSn films with 20% content were epitaxied at 170 °C, with their thickness ranging from 7 to 50 nm.

## Result

**Figure 1:**
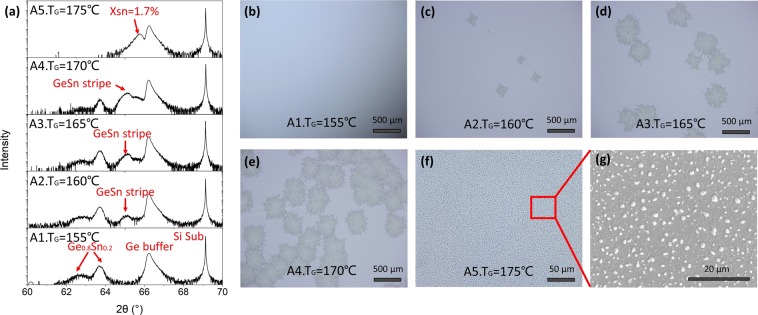
XRD and microscope results of samples in Series A. (**a**) HR-XRD (004) “ω-2θ” scanning of samples in Series A, which were grown at increasing growth temperature, with their corresponding microscope images displayed in (**b**) to (**f**); and (**g**) is the SEM image of the red box in (**f**).

Series A samples were prepared to investigate the influence of growth temperature towards the surface morphology. Briefly, 50 nm GeSn films were grown on Ge buffered Si substrate via MBE (Molecular Beam Epitaxy) method at growth temperature varied from 155 to 170 °C, and the deposition rate of Ge and Sn were keep constant as r_Ge_ = 0.06 nm/s and r_Sn_ = 0.015 nm/s during the epitaxial progress of GeSn films (Table 1). Due to the characteristics of MBE method that the temperature of substrate would have negligible influence to the beam flow from source material, so the Ge and Sn deposition rate were constant while the substrate temperature range from 155 to 175 °C. The XRD results, microscope images and SEM image of series A samples are as shown in Fig. 1. For GeSn samples prepared at 155 °C (Sample A1), only the peaks of strain and partly relaxed GeSn, Ge buffer and Si substrate are observed in XRD curves (Fig. 1a), different from other material, the relaxation of GeSn film prepared via MBE method appears as the emergence of a new peak with lower diffraction angle, rather the the shift of peak positon of the film^24^ in its (004) XRD curves, and a SIMS (Secondary Ion Mass Spectroscopy) test had been conducted for sample A1 and its results confirms that the Sn content in GeSn film is 20% and homogeneous. (S1 in Supplementary Information), the Sn content is calculated to be 20.3% via the peak position, which agrees well with the result extracted from the Ge: Sn ratio and SIMS results. Figure 1b is the microscope image of sample A1, the surface is clear and smooth, no segregated Sn or other micro structure are observed on the surface. However, when the growth temperature of Ge_0.8_Sn_0.2_ raised to 160 °C (sample A2), a new peak at 65.16 ° appears in the XRD curve, and stripe with length ranging from 50–100 μm are formed on the surface (Fig. 1c). Further increase of growth temperature leads to the fade of Ge_0.8_Sn_0.2_ peaks and the enhancement of peak at 65.16 ° (sample A3 and A4), accompanied by the raise of the length and density of stripes (Fig. 1d,e). Especially for sample A4, the Ge_0.8_Sn_0.2_ peaks almost disappear, and nearly the whole surface is covered by stripes. The evolution of XRD curves and surface morphology with growth temperature shows clear regularity and relevance, a positive correlation can be confirmed between the peak intensity at 65.16 ° and the occupation area of stripes. This correlation indicates that the peak at 65.16 ° is signal from stripes, and the stripes are single crystalline GeSn with 5% Sn content. When the growth temperature increased to 175 °C (sample A5), the Ge_0.8_Sn_0.2_ film segregated completely, confirmed by the disappearance of Ge_0.8_Sn_0.2_ peaks in XRD curves and the coverage of segregated Sn on the surface (Fig. 1f,g). A new peak with position at 65.75 ° appears, indicating that there are still 1.7% Sn atom remained in Ge atom matrix while most of Sn in Ge_0.8_Sn_0.2_ film segregates to the surface. Meanwhile, it can be noticed that there is a bump on the right of GeSn stripe peaks in the XRD result of sample A4, which is supposed to be caused by the segregated area in the center of GeSn stripe patterns, the specific discussion can been seen in S2 in the Supplementary Information. Based on the above results, it can be found that excessive growth temperature (175 °C) led to the completely segregation of Ge_0.8_Sn_0.2_ film while no segregated Sn or microstructure was formed on the Ge_0.8_Sn_0.2_ film surface prepared under low growth temperature (155 °C). The formation of GeSn stripes only happened in a small range of growth temperature of Ge_0.8_Sn_0.2_ film and the occupation area is directly proportional to the growth temperature, so the formation of GeSn stripes can be considered to be caused by the partly segregation of Ge_0.8_Sn_0.2_ film.

**Figure 2:**
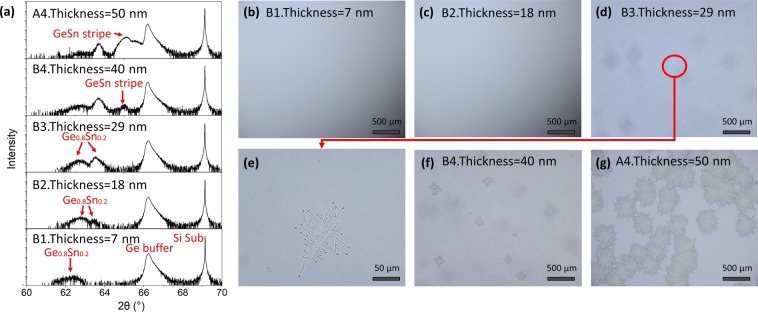
XRD and microscope results of samples in Series B. (**a**) HR-XRD (004) “ω-2θ” scanning of samples in Series B, the Ge_0.8_Sn_0.2_ films were grown under 170 °C, with thickness ranging from 7 to 50 nm, and (**b**) to (**g**) are their corresponding microscope images.

To investigate the morphology evolution with film thickness, Ge_0.8_Sn_0.2_ films with thickness ranging from 7 to 50 nm were grown on Ge buffered Si substrates at 170 °C (Series B samples). Figure 2a is the (004) XRD scanning curves of Series B samples and their corresponding microscope images are as shown in Fig. 2b–g. For sample B1 with thickness of 7 nm, the three peaks from left to right in the (004) XRD results correspond to the strained Ge_0.8_Sn_0.2_ film, Ge buffer and Si substrate, respectively. The intensity of Ge_0.8_Sn_0.2_ XRD peak is very low due to the small thickness (7 nm) of the film. The surface of sample B1 is clear and smooth, no segregated Sn or other micro structure is observed on the surface (Fig. 2b). Continuous growth of Ge_0.8_Sn_0.2_ film led to the appearance of a new peak on the right of strained Ge_0.8_Sn_0.2_ peak in the (004) XRD curve. Considering the constant deposition rate of Ge and Sn, and no segregated Sn is observed on the surface (Fig. 2c), the newly appeared peak is supposed to be caused by the relaxation of Ge_0.8_Sn_0.2_ film. When the thickness of Ge_0.8_Sn_0.2_ film reached to 29 nm (sample B3), GeSn stripes with length of ~50 μm formed on the surface (Fig. 2d,e), while no new peak related to GeSn stripes is observed in the (004) XRD curves, which may caused by the smaller occupation area of GeSn stripes. Further deposition of Ge_0.8_Sn_0.2_ film leads to the increase of density and length of GeSn stripes (Fig. 2f,g), causing the appearance and enhancement peak corresponded to GeSn stripes and the fade of peaks related to Ge_0.8_Sn_0.2_ film. The above results indicate that the formation of GeSn stripes in series B samples occurred after the relaxation of Ge_0.8_Sn_0.2_ film. A positive proportional relationship is confirmed between the length of the GeSn stripes and the film thickness (Growth Time), and the advanced velocity of GeSn stripe patterns is calculated as ~50 nm/s.

**Figure 3:**
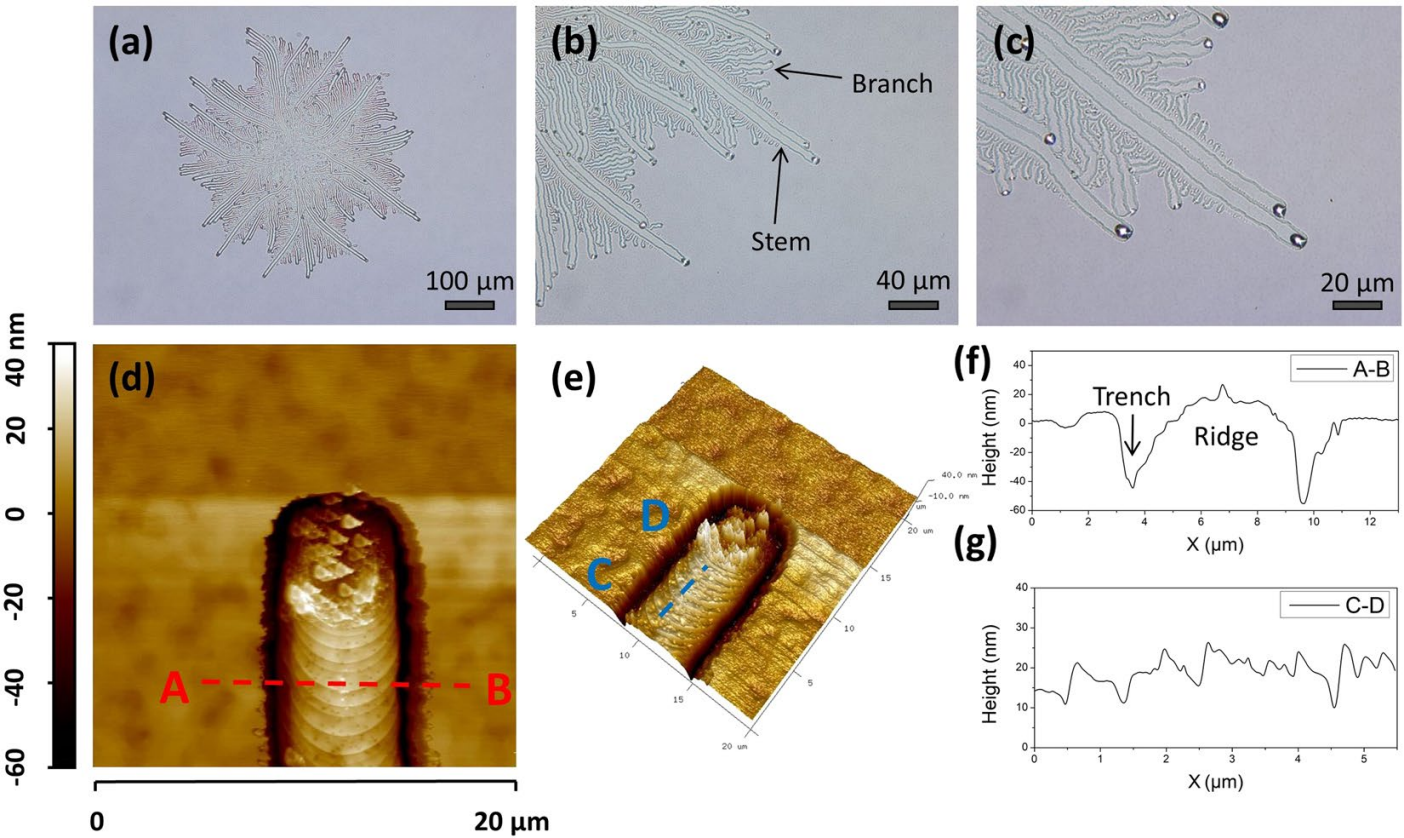
Microscope and AFM results of samples A4. (**a**) Microscope image of GeSn stripes, with its magnified images displayed in (**b,c**). (**d,e**) are 2D and 3D AFM images of a single GeSn stripe, respectively. The Sn droplets at the head of the stripe is removed by HCl solution. (**f,g**) are line profile of red line ‘AB’ and blue line ‘CD’ in (**d,e**), respectively.

Figure 3a–c are microscope images of GeSn stripes in sample A4 (50 nm Ge_0.8_Sn_0.2_ film grown at 170 °C). It shows two kind of stripes – the larger and longer ones that extend from the center of the patterns, which are called ‘stem’, the shorter and narrower ones that extend out from the ‘stem’, which are called ‘branch’, white spots can be found at the end of stripes, which are supposed to be the Sn droplets. The 2D and 3D AFM images (Fig. 3d,e) show the complex structure of a GeSn stripe at the end, while the suspected Sn droplets at the end of the stripe are removed by HCl solution in advance. The AFM line profile in Fig. 3f reveals that the center of stripe, which is called as ridge, is about 30 nm above the surrounding Ge_0.8_Sn_0.2_ film, and two 50 nm deep trenches divide the stripe from the surrounding Ge_0.8_Sn_0.2_ film. Another prominent features are bundle of ripple-like lines along the stripes, which are confirmed to be caused by the height fluctuation with a period of 1 μm via the AFM line profile in Fig. 3g. The presence of ridge and trench indicate that the formation of GeSn stripes is caused by the migration of Sn droplets, and the ripple-like lines along the stripes demonstrate that advance of the Sn droplets may be discrete with step length of 1 μm.

**Figure 4:**
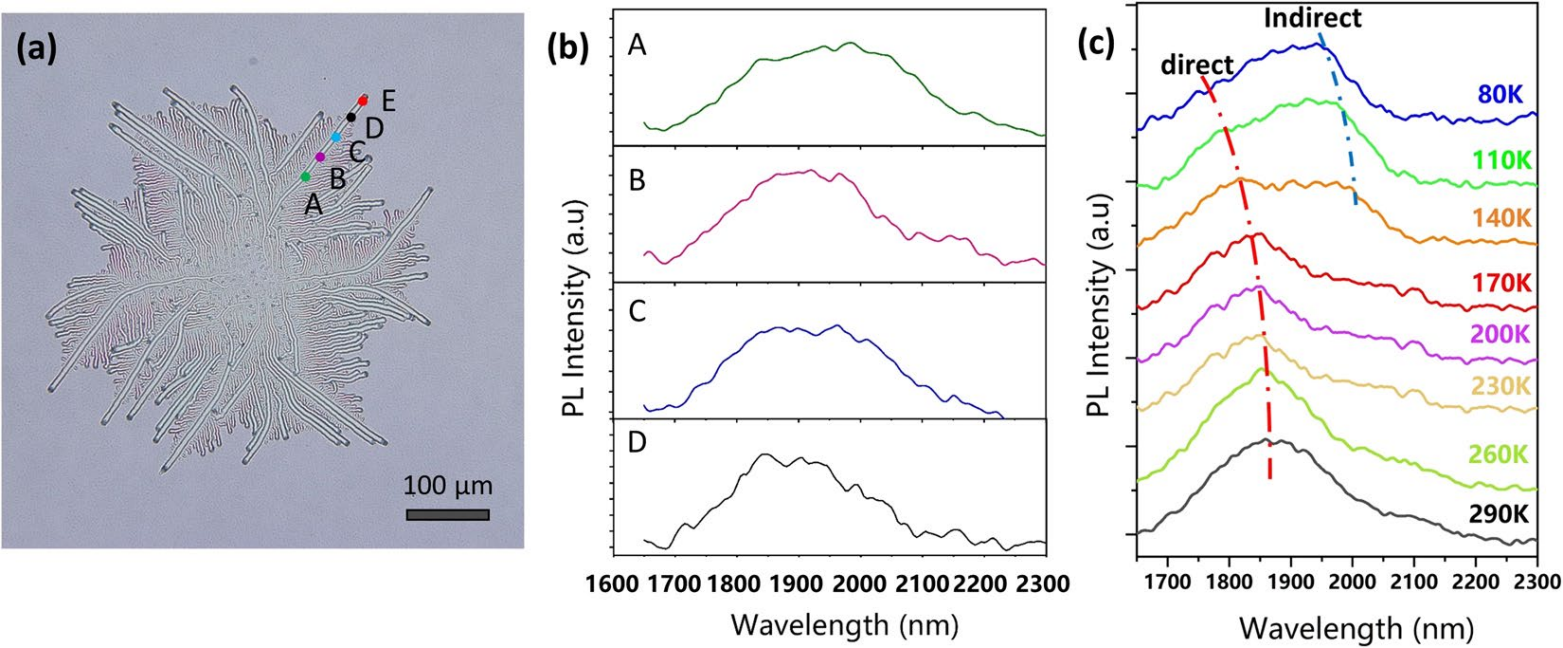
Photoluminescence results. (**a**) The microscope image of GeSn stripe patterns for photoluminescence (PL) test. The incident light was focused into a spot of ~20 μm using a 15× objective, and A, B, C,D and E are the positions of focused light spot in the course of PL test, and the PL results of position A, B, C and D at room temperature (300 K) are plotted in (**b**). (**c**) The temperature-dependent PL results of position E.

A confocal microscope equipped with a 785 nm solid-state laser was used to characterize the optical properties of GeSn stripe by photoluminescence (PL), and point A, B, C,D and E in Fig. 4a are the positions of incident light spots in the course of the PL test, Fig. 4b is the PL results measured at room temperature (290 k) for position A,B, C and D. Clear PL peaks with relatively high signal to noise ratio is achieved in the results of position A to D, indicating the low defect density and high crystalline quality in the GeSn stripes. Nearly the same peak position around 1900 nm for A to D agrees well with the bandgap calculated by the XRD result in Fig. 2a (0.65 eV), demonstrating the uniform Sn content along the GeSn stripe. Meanwhile, a temperature-dependent PL test were performed at position E and its results are plotted in Fig. 4c. There are two peaks in the PL results, the high-energy one, which originates from direct bandgap transition, is visible within the temperature range of 290 K to 110 K, and the low-energy one, which originates from indirect bandgap transition, can be distinguished in the temperature range from 80 K to 140 K. With the decreasing temperature, both the direct and indirect peaks experience a blue shift caused by the narrowing of bandgap. For direct peaks, the peak intensity decreased with decreasing temperature while peak intensity of the indirect ones increased instead, this can be explained by the fact the less electron occupation of the τ-valley as a result of suppressed thermal excitation from the lower L-valley at the low temperature. Compared to the works that had been published^25,26^, the PL peak position of GeSn stripe is reasonable compared to the 1800 nm for 4% Sn content GeSn film and 1980 nm for 6% Sn content GeSn film, and the intensity of the direct PL peaks decreases and the indirect PL peaks increase with decrease temperature in this work, just as the results of GeSn films in previous work.

**Figure 5:**
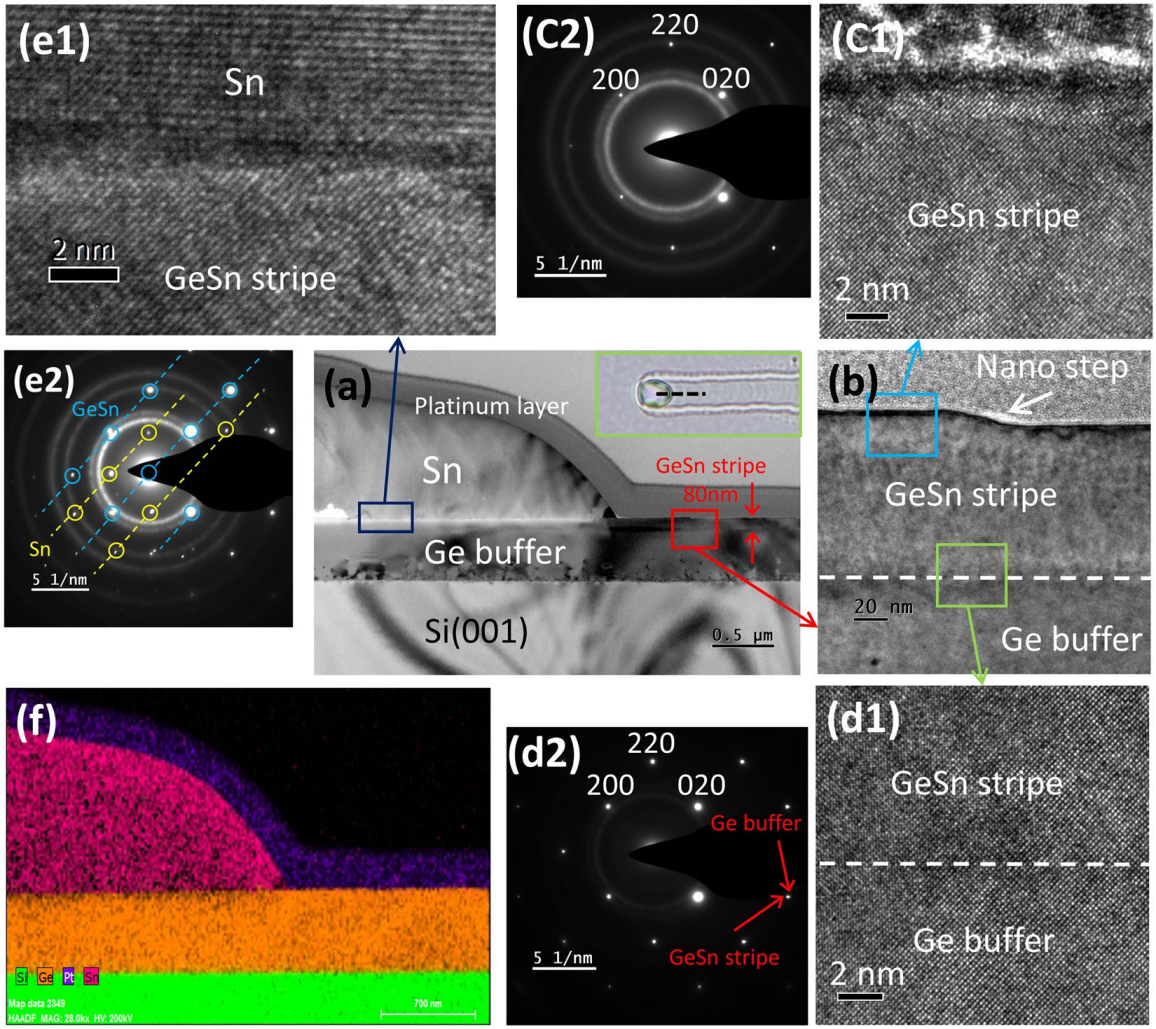
The inset in (**a**) is the microscope image of a GeSn stripe, which is milled by FIB along the black line to expose the axial cross-section for further characterization, (**a**,**f**) are the TEM image and EDS element mapping of the above mentioned cross-section, respectively. (**b**) Magnified TEM image of interfacial area between the GeSn stripe and Ge buffer. (**c1**) The HRTEM image of the GeSn stripe surface, with its SAED pattern shown in (**c2**). (**d1**) The HRTEM image of the interface between GeSn stripe and Ge buffer, with its SAED pattern shown in (**d2**). (**e1**) The HRTEM image of interfacial area between Sn droplet and GeSn stripe, with its SAED pattern shown in (**e2**).

The crystalline quality of GeSn stripe, the interface between Sn droplet and GeSn stripe, as well as the epitaxial relationship between Ge buffer and GeSn stripe are characterized by high-resolution transmission electron microscope (HRTEM). The sample was first capped by a platinum layer, then milled by focus-ion-beam (FIB) to expose the cross-section along the black line in the inset of Fig. 5a for further investigation. Figure 5a is the low-magnification TEM image with its corresponding EDS mapping shown in Fig. 5f. The pink, orange and green spot in Fig. 5f represent the distribution of Sn, Ge and Si element, the accumulation of pink spots at the end of GeSn stripe confirms that the white spots observed in Fig. 3c are Sn droplets, the average Sn content in the stripe extracted from EDS results is about 5% and is in conformance with the results calculating from XRD peak position. In Fig. 5a, no threading dislocation is observed in the stripe area, demonstrating the high crystalline quality. The thickness of GeSn stripe is measured to be 80 nm, which is 30 nm higher than the initial Ge_0.8_Sn_0.2_ film, agreed well with the AFM line profile in Fig. 3f. It is also found that the thickness of GeSn stripe under the Sn droplet decreases along the extension direction, confirming that the Ge_0.8_Sn_0.2_ material at the interface of Sn droplets and GeSn stripes is consumed for GeSn stripe production. Figure 5b is the magnified TEM image of the interfacial area between GeSn stripe and Ge buffer, no clear boundary between GeSn stripe and Ge buffer is observed. A 20 nm high nano step is found at the surface of the stripe, which attributed the ripple-like line observed in Fig. 3d,e. Figure 5c1 and d1 are HRTEM images of the GeSn stripe surface and the interface between GeSn stripe and Ge buffer, with their SAED pattern displayed in Fig. 5c2 and d2. A coherent interface was formed between GeSn stripe and Ge buffer along the <001> plane (Fig. 5d1), and the SAED results in Fig. 5c2 and d2 further confirm the GeSn stripe is single crystal with a diamond cubic structure, and the epitaxial relationship between the GeSn stripe and Ge buffer. Figure 5e1 is HRTEM image of the interface of Sn droplets and GeSn stripe, with its SEAD pattern shown in Fig. 5e2. The SEAD results reveal two sets of spots that corresponded to the GeSn stripe and the Sn, the GeSn stripe under the Sn droplets remains as a single crystal with a diamond cubic structure, whereas the Sn droplets presents a β-phase (body-centered tetragonal) single crystal, the different atomic arrangement at the interface (Fig. 5e1) also support the SEAD results in Fig. 5e2.

## Discussion

**Figure 6:**
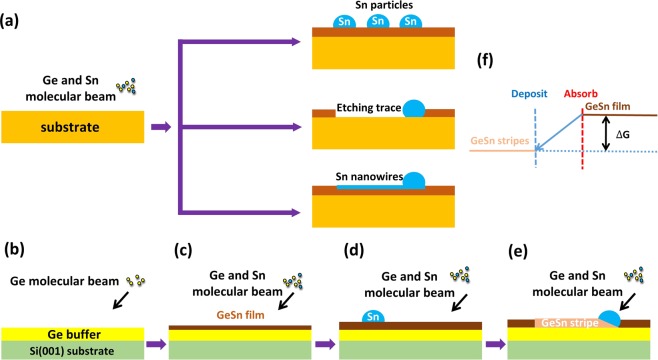
(**a**) The schematic illustration of the Sn segregation phenomenon of low Sn content GeSn films observed in previous works. (**b–e**) The schematic illustration of the formation progress of GeSn stripe during the epitaxy of Ge_0.8_Sn_0.2_ film. (**f**) is the diagram showing the difference in free energy between Ge_0.8_Sn_0.2_ film and stripe, which is the driving force for the progression of the GeSn stripe growth.

As a promising group IV material, GeSn has attracted a lot of attention and several work had been done to investigate the Sn segregation during the epitaxial progress of GeSn film with low Sn content (5–18%) aiming at figuring out the mechanism behind the segregation of Sn and thus optimizing the epitaxial technique, it’s has been found out that beside the epitaxy breakdown caused by the accumulation of defect, the high growth temperature and the strain relaxation of GeSn film would lead to the segregation of Sn in GeSn film during epitaxial progress, and the behavior of segregated Sn on GeSn film can classified into 3 types (Fig. 6a): (1) The segregated Sn leave stand still Sn particles on the surface^20,21^; (2) The segregated Sn accumulates as Sn droplets, moving on the surface of GeSn film and etching the material it passed by, leaving etching traces behind^21^; (3) The segregated Sn accumulates into Sn droplets, moving on the surface of GeSn film and leave Sn wires behind^22^. It’s the energy gap between GeSn before and after segregation, the growth temperature, and the strain in GeSn film determine the driving energy of Sn droplets and thus determine the behavior of segregated Sn. If the driving energy of segregated Sn is too small to drive the Sn droplets moving on the surface, the segregated Sn tends to leave motionless Sn particles on the film, while the drive force is bigger enough to drive Sn droplets moving on the surface, the moving Sn droplets would etching the GeSn material it passed by or leave Sn wires behind.

In this work, Series A samples stated clearly that no GeSn stripes would form on the surface at low epitaxial temperature, and the formation of GeSn stripes is lead by the moving of segregated Sn. A positive proportion can be confirmed between the occupation area and the growth temperature, indicating the formation of GeSn stripe is related to high epitaxial temperature of Ge_0.8_Sn_0.2_ film. Meanwhile, Series B samples made it clear that the formation of GeSn stripe occurred after the relaxation of Ge_0.8_Sn_0.2_ film, demonstrating that the formation of GeSn stripes has a connection with the relaxation of Ge_0.8_Sn_0.2_ film. Here, combining the results extract from Series A,B samples and previous work, it can be found that the formation of GeSn stripes is originate from the segregated Sn caused by excessive epitaxial temperature and the relaxation of GeSn film, just as the Sn segregation phenomenon observed in previous work. However, in this work, different from the results in previous work that segregated Sn stand still or move and leaving etching trace or Sn wires on the surface of GeSn film, the segregated Sn behave in a new ways, moving on the surface of Ge_0.8_Sn_0.2_ film and converting the Ge_0.8_Sn_0.2_ material it passed by and producing GeSn stripe behind. Here, the different behavior of segregated Sn come from the huge Gibbs free energy gap between the GeSn films before and after Sn segregation. The Gibbs free energy change during the Sn segregation progress can be wrote as ∆G = −(∆G_V_ + ∆G_S_) + ∆G_in_, where ∆G_V_ is the reduction of the free energy from GeSn films to segregated Ge and the derivative (in this work are GeSn stripes and in previous work are motionless Sn particles, Sn wires or Sn etch trace), ∆G_S_ is the decrease of strain energy caused by the conversion from GeSn film to GeSn stripe (this work) and segregated Ge (previous work), and ∆G_in_ is the interfacial energy caused by the trenches formed beside the GeSn stripes (this work) or Sn etching trace and Sn wires (previous work). Due to the high Sn content (20%) in this work, the ∆G_V_ and ∆G_S_ are big enough to drive the movement of Sn droplets as well as converting the Ge_0.8_Sn_0.2_ film to GeSn stripe at the same time (Fig. 6f). It’s the high Sn content in Ge_0.8_Sn_0.2_ film generate large enough Gibbs free energy gap and thus make the segregated Sn behave in a new way. According the above results, the formation of GeSn stripes during the epitaxial progress of Ge_0.8_Sn_0.2_ film can be described as follows: (1) 250 nm Ge buffer is deposited on Si(001) substrate (Fig. 6b); (2) the epitaxy of Ge_0.8_Sn_0.2_ film is started (Fig. 6c); (3) as the progress of Ge_0.8_Sn_0.2_ film growth, the thickness of Ge_0.8_Sn_0.2_ film reaches its critical thickness (between 7 and 18 nm in this work) and gets relaxed. Further deposition of Ge_0.8_Sn_0.2_ film leads to the accumulation of defects and the degeneration of the film, causing the segregation of Sn on the surface (Fig. 6d); (4) The segregated Sn accumulates as Sn droplet and migrates on the surface, it takes in Ge_0.8_Sn_0.2_ film it passed by, and produces high quality GeSn stripe with 5% Sn content behind (Fig. 6e).

The formation process of GeSn stirpes during the epitaxy of Ge_0.8_Sn_0.2_ film, from micro terms, is that the segregated Sn acts as catalyst droplets, converting the Ge_0.8_Sn_0.2_ film to GeSn stripe via the strain relaxation mechanism, and it’s similar to the in-plane solid-liquid-solid (IPSLS) growth mode for Si nanowires synthesis reported by Yu^27,28^, the difference is that the precursor film and metal catalyst droplets in this work are prepared at the same time during the epitaxy of Ge_0.8_Sn_0.2_ film via strain relaxation mechanism. However, from the macro prospective, the formation of GeSn stripes can be seemed as GeSn with low Sn content participates from Ge_0.8_Sn_0.2_ film, which means that the high Sn content GeSn film can spontaneously convert to high crystalline quality GeSn stripe with low Sn content during molecular beam epitaxy under certain growth temperature, and this gives a positive meaning to the Sn segregation phenomenon, which is harmful and hated before^29,30^.

In summary, two series of Ge_0.8_Sn_0.2_ samples were grown on Ge buffered Si substrates to investigate the influence of growth temperature and film thickness towards the morphology evolution of the surface, and it is found out that the over-temperature growth and the strain relaxation of Ge_0.8_Sn_0.2_ film would lead to the formation of GeSn stripes on the surface. The results for XRD, microscope, AFM, PL and TEM confirm that the stripes are high quality single crystalline GeSn with 5% Sn content, and the formation of the GeSn stirpes is guided by the segregation and migration of Sn droplets via the over-temperature growth and strain relaxation of Ge_0.8_Sn_0.2_ film. The formation of GeSn stripes reported in this work, from one hand, is a novel Sn segregation phenomena that can help us to better understand the interaction between the Sn and Ge atoms during epitaxy and optimize the growth technique. On the other hand, it proposes an effective strategy to fabricate high crystalline quality self-assembled GeSn stripe on Si using Ge_0.8_Sn_0.2_ film as precursor without the need of extra step to prepare metal droplet catalyst, and has great potential for future optoelectronic and microelectronic applications.

## Methods

### Epitaxial Growth

All samples in this work were grown on 4 inch n-type Si(001) wafer (resistivity:1–10 Ω) using a solid source MBE chamber equipped with a Ge e-beam evaporator and Sn pyrolytic BN effusion cell for Ge and GeSn film deposition. The Si substrates was first cleaned via RCA method and then loaded into an ultra-high-vacuum chamber, after the degassing at 300 °C, the Si substrates was transferred to growth chamber and heated at 850 °C for deoxidization. After that, 250 nm Ge buffer layer was deposited on the Si substrate, followed by five times cycle annealing to improve the crystalline quality in Ge buffer layer. Finally, Ge_0.8_Sn_0.2_ films with constant deposition rate of r_Ge_ = 0.06 nm/s and r_Sn_ = 0.015 nm/s were grown under 155 to 175 °C.

### Characterization

The XRD test was performed with PANalytical X’Pert MRD XL diffractometer at ambient environment. The AFM images were taken with AFM (DimensionEdge). Photoluminescence (PL) was conducted with a system consisting of a continuous wave laser of 785 nm with a maximum optical power of 2.5 W, a 15X Thorlabs reflective objective (NA = 0.3), a spectrometer whose f = 300 mm (Princeton Instrument SP-2300), an InGaAs photomultiplier tube (PMT) whose cutoff wavelength is 2400 nm and a Stanford SR830 lock-inamplifier. The TEM images were taken with a high-resolution TEM (Tecnai G2 F20 S-Twin) operating at electron beam energy of 200 KeV, and the energy-dispersive X-ray spectroscopy (EDS) measurement was taken with the aforementioned TEM under TEM model.

## Date availability

The datasets generated during and/or analysed during the current study are available from the corresponding author on reasonable request.

## Supporting information

Supplementary information.

## References

[1] Soref R (2007). The Past, Present, and Future of Silicon Photonics. IEEE J. Quantum Electron..

[2] Soref RA (1993). Silicon-Based Optoelectronics. Proc. IEEE.

[3] Sturm JC (1998). Advanced column-IV epitaxial materials for silicon-based optoelectronics. MRS Bull..

[4] von den Driesch N (2018). Advanced GeSn/SiGeSn Group IV Heterostructure Lasers. Adv. Sci..

[5] Ghetmiri SA (2017). Study of a SiGeSn/GeSn/SiGeSn structure toward direct bandgap type-I quantum well for all group-IV optoelectronics. Opt. Lett..

[6] Tran H (2018). High performance Ge0.89Sn0.11 photodiodes for low-cost shortwave infrared imaging. J. Appl. Phys..

[7] Yang F (2019). Highly Enhanced SWIR Image Sensors Based on Ge1–xSnx–Graphene Heterostructure Photodetector. ACS Photonics.

[8] Huang BJ, Lin JH, Cheng HH, Chang GE (2018). GeSn resonant-cavity-enhanced photodetectors on silicon-on-insulator platforms. Opt. Lett..

[9] Wirths S (2015). Lasing in direct-bandgap GeSn alloy grown on Si. Nat. Photon..

[10] Cong H (2018). Multilayer Graphene-GeSn Quantum Well Heterostructure SWIR Light Source. Small.

[11] Chrétien J (2019). GeSn Lasers Covering a Wide Wavelength Range Thanks to Uniaxial Tensile Strain. ACS Photonics.

[12] Sau JD, Cohen ML (2007). Possibility of increased mobility in Ge-Sn alloy system. Phys. Rev. B.

[13] Chou C, Lin Y, Wu Y (2018). Implementing P-Channel Junctionless Thin-Film Transistor on Poly-Ge0.95Sn0.05 Film Formed by Amorphous GeSn Deposition and Annealing. IEEE Electron Device Lett..

[14] Huang Y (2018). Vertically Stacked Strained 3-GeSn-Nanosheet pGAAFETs on Si Using GeSn/Ge CVD Epitaxial Growth and the Optimum Selective Channel Release Process. IEEE Electron Device Lett..

[15] Liu T (2018). High-Mobility GeSn n-Channel MOSFETs by Low-Temperature Chemical Vapor Deposition and Microwave Annealing. IEEE Electron Device Lett..

[16] Marris-Morini D, Vakarin V, Ramirez JM (2018). Germanium-based integrated photonics from near- to mid-infrared applications. Nanophotonics.

[17] Pukite PR, Harwit A, Iyer SS (1989). Molecular beam epitaxy of metastable, diamond structure SnxGe1−xalloys. Appl. Phys. Lett..

[18] Thurmond CD, Trumbore FA, Kowalchik M (1956). Germanium Solidus Curves. The Journal of Chemical Physics.

[19] Eaglesham DJ (1995). Semiconductor molecular‐beam epitaxy at low temperatures. J. Appl. Phys..

[20] Kormoš L (2017). Surface analysis of epitaxially grown GeSn alloys with Sn contents between 15% and 18%. Surf. Interface Anal..

[21] Tsukamoto T (2015). Investigation of Sn surface segregation during GeSn epitaxial growth by Auger electron spectroscopy and energy dispersive x-ray spectroscopy. Appl. Phys. Lett..

[22] Deng X, Yang BK, Hackney SA, Krishnamurthy M, Williams DRM (1998). Formation of Self-Assembled Quantum Wires during Epitaxial Growth of Strained GeSn Alloys on Ge(100): Trench Excavation by Migrating Sn Islands. Phys. Rev. Lett..

[23] Wang W, Li L, Tok ES, Yeo Y-C (2015). Self-assembly of tin wires via phase transformation of heteroepitaxial germanium-tin on germanium substrate. J. Appl. Phys..

[24] Wang W, Zhou Q, Dong Y, Tok ES, Yeo Y-C (2015). Critical thickness for strain relaxation of Ge1−xSnx (x ≤ 0.17) grown by molecular beam epitaxy on Ge(001). Appl. Phys. Lett..

[25] Ghetmiri SA (2014). Direct-bandgap GeSn grown on silicon with 2230 nm photoluminescence. Appl. Phys. Lett..

[26] Du W (2014). Competition of optical transitions between direct and indirect bandgaps in Ge1−xSnx. Appl. Phys. Lett..

[27] Yu L, I Cabarrocas PR (2010). Growth mechanism and dynamics of in-plane solid-liquid-solid silicon nanowires. Phys. Rev. B.

[28] Yu L (2014). In-plane epitaxial growth of silicon nanowires and junction formation on Si(100) substrates. Nano Lett..

[29] Johll H (2015). Influence of hydrogen surface passivation on Sn segregation, aggregation, and distribution in GeSn/Ge(001) materials. J. Appl. Phys..

[30] Chambouleyron I, Marques FC (1989). Use of hydrogenation in the study of the properties of amorphous germanium tin alloys. J. Appl. Phys..

