# Supplementary material for: Spontaneously Conversion from Film to High Crystalline Quality Stripe during Molecular Beam Epitaxy for High Sn Content GeSn
Source: Sci Rep. 2020 Apr 9;10:6161. doi: 10.1038/s41598-020-63152-y (PMC7145829; doi:10.1038/s41598-020-63152-y)
Supplement: Supplementary file 1 — Supplementary information. [file 41598_2020_63152_MOESM1_ESM.pdf]

Supplementary Information

Spontaneously Conversion from Film to High Crystalline Quality Stripe during Molecular Beam Epitaxy for High Sn Content GeSn

Nan Wang[1,2], Chunlai Xue*[1,2],Fengshuo Wan[1,2],Yue Zhao[1,2], Guoyin Xu[1,2], Zhi Liu[1,2], Jun Zheng[1,2], Yuhua Zuo[1,2],Buwen Cheng[1,2] and Qiming Wang[1,2]

1. State Key Laboratory on Integrated Optoelectronics, Institute of Semiconductors, Chinese Academy of Sciences, Beijing 100083, P. R. China.
2. Center of Materials Science and Optoelectronics Engineering, University of Chinese Academy of Sciences, Beijing 100049, China.

Corresponding Author:
*E-mail: clxue@semi.ac.cn

Contents:

S1: The SIMS results of sample A1

S2: The analysis to the XRD results of sample A4

S1：The SIMS results of sample A1

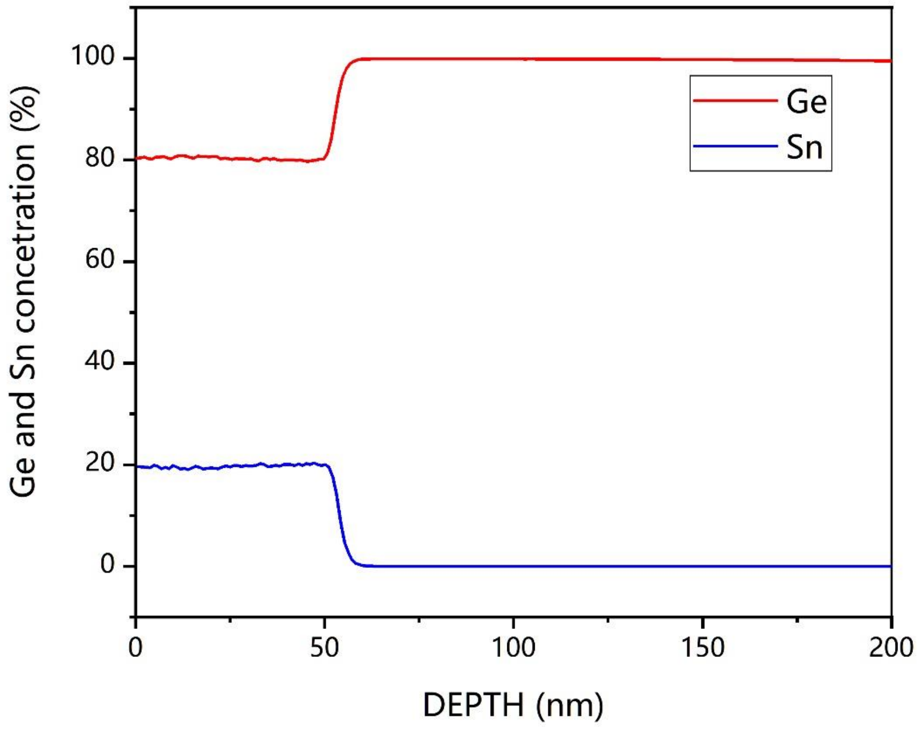

FigureS1. The SIMS result of sample A1, the blue and red line represent the distribution of Sn and Ge element.

The SIMS results of sample A1 (**Figure S**1) indicate that the Sn content in $Ge_{0.8}Sn_{0.2}$ film is homogeneous and the two GeSn peaks in the XRD results of sample A1 (Figure 1a in the manuscript) are strain and partly relaxed peak of $Ge_{0.8}Sn_{0.2}$ film.

S2: The analysis to the XRD results of sample A4

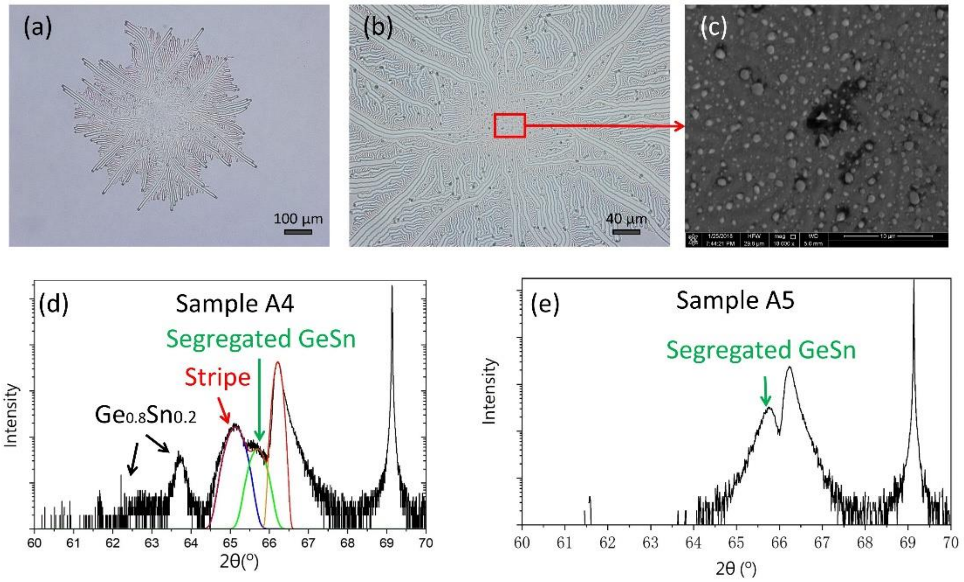

Figure S2. (a) The microscope image of stripes on sample A4, with its magnified image displayed in (b), and (c) is the SEM image of the red box in (b), (d) and (e) are the (004) XRD results of sample A4 and A5.

Figure S2a is the microscope image of GeSn stripes pattern in sample A4, with its magnified image shown in **Figure S2**b and c. It's found out the center of the pattern is segregated, with Sn dots cover the center area. In the XRD results of sample A5 (**Figure S2**e), it's confirmed that there was still 1.7% Sn remained in the Ge matrix and the (004) XRD peak position corresponding the segregated $Ge_{0.8}Sn_{0.2}$ film is around the 65.75 ^{o} when the film is fully segregated. Meanwhile, the peak between the $Ge_{0.8}Sn_{0.2}$ peak and Ge buffer peak is the superposition of two peaks with position of 65.16 ^{o} and 65.75 ^{o} in the XRD results of sample A4, just as shown in **Figure S2**d. Considering all the above results, the bump on the right GeSn stripe peak in **Figure S2**d is arising from the segregated area in the center of GeSn stripe patterns, rather than heterogeneous Sn content in the GeSn stripe.
